# High Prevalence of *TERT* and *CTNNB1* Mutations in Brazilian HCC Tissues: Insights into Early Detection and Risk Stratification

**DOI:** 10.3390/ijms26136503

**Published:** 2025-07-06

**Authors:** Thaís Barbosa Ferreira Sant’Anna, Mariana Leonardo Terra, Jose Junior França de Barros, Leonardo Alexandre de Souza Ruivo, Arlete Fernandes, Maria Dirlei Ferreira de Souza Begnami, Vera Lucia Nunes Pannain, Antônio Hugo José Fróes Marques Campos, Otacilio da Cruz Moreira, Natalia Motta de Araujo

**Affiliations:** 1 Laboratory of Molecular Virology and Parasitology, Oswaldo Cruz Institute, Oswaldo Cruz Foundation, Rio de Janeiro 21040-900, Brazil; thaisbfsantanna@gmail.com (T.B.F.S.); marianaterra.biomed@gmail.com (M.L.T.); barros@ioc.fiocruz.br (J.J.F.d.B.); leonardo.ras@gmail.com (L.A.d.S.R.); otacilio@ioc.fiocruz.br (O.d.C.M.); 2 Department of Pathology, Clementino Fraga Filho University Hospital, Federal University of Rio de Janeiro, Rio de Janeiro 21941-617, Brazil; ladbatera@gmail.com (A.F.); verapannain@gmail.com (V.L.N.P.); 3 Department of Anatomic Pathology, A.C.Camargo Cancer Center, São Paulo 01509-010, Brazil; mariadirlei@gmail.com; 4 Sírio-Libanês Institute of Education and Research, Sírio-Libanês Hospital, São Paulo 01308-050, Brazil; 5 A.C.Camargo Biobank, A.C.Camargo Cancer Center, São Paulo 01509-010, Brazil; antonio.mcampos@rededor.com.br; 6 D’Or Institute for Research and Education, IDOr, São Paulo 01401-002, Brazil

**Keywords:** liver carcinogenesis, telomerase activation, β-catenin signalling, mutation profiling, tumour biomarkers, viral hepatitis, gene expression analysis, Latin America

## Abstract

Hepatocellular carcinoma (HCC) is a major cause of cancer-related mortality worldwide, but its molecular drivers remain underexplored in Latin American populations. This study investigated the prevalence, co-occurrence, and tissue distribution of somatic mutations in the *TERT* promoter (C228T/C250T) and *CTNNB1* exon 3, as well as *TERT* gene expression, in liver tissues from Brazilian patients. A total of 85 samples (42 HCC, 21 cirrhosis, and 22 hepatitis) were analysed using Sanger sequencing and RT-qPCR. *TERT* promoter mutations were detected in 47.6% of HCC tissues, including C228T (45.2%) and C250T (2.4%), and were also present in 19% of cirrhotic tissues but absent in hepatitis samples, supporting their emergence in early hepatocarcinogenesis. *CTNNB1* exon 3 mutations occurred in 17.2% of HCCs and significantly co-occurred with *TERTp* mutations (66.7%, *p* = 0.0485), although the number of *CTNNB1*-mutated cases was limited. *TERT* expression was significantly upregulated in HCC tissues regardless of mutation status (*p* < 0.001). This is the first study to characterise these mutations in Brazilian HCC patients, highlighting the C228T mutation as a promising biomarker for early detection and molecular surveillance in cirrhotic individuals. Despite the genetic admixture of the studied population, the mutational patterns were comparable to those reported in more homogeneous populations, reinforcing the global relevance of these molecular alterations.

## 1. Introduction

Hepatocellular carcinoma (HCC) is the most common type of liver cancer, accounting for approximately 90% of primary liver malignancies. Due to its aggressive nature and poor prognosis, the global incidence of HCC closely mirrors patient mortality, representing a major public health challenge [1,2,3]. Currently, liver cancer is the sixth most common cancer and the third leading cause of cancer-related deaths worldwide [4,5]. Despite advances in medical treatment, HCC prognosis remains unfavourable, with a 5-year survival rate below 20%, primarily due to late diagnosis [2]. Early detection is crucial to improving patient outcomes, yet this remains challenging, particularly in populations with limited healthcare access or inadequate screening protocols.

Globally, HCC arises from a complex interplay of genetic factors, environmental exposures, viral infections, and chronic liver diseases [6]. Major risk factors include chronic hepatitis B virus (HBV) or hepatitis C virus (HCV) infections, chronic alcohol consumption, and metabolic dysfunction-associated steatotic liver disease (MASLD), formerly known as non-alcoholic fatty liver disease (NAFLD), frequently accompanied by obesity and type 2 diabetes. These risk factors promote chronic hepatic inflammation, leading to repeated cycles of hepatocyte injury, regeneration, and eventual cirrhosis, observed in 80–90% of HCC cases [1].

Among the diverse genetic alterations associated with HCC, somatic mutations in the telomerase reverse transcriptase promoter (*TERTp*) and in the β-catenin-encoding *CTNNB1* gene are particularly noteworthy [7,8]. *TERTp* mutations (notably C228T, also referred to as −124C > T, and C250T, also referred to as −146C > T) increase telomerase activity, critical for tumour cell immortality [9]. A recent comprehensive review revealed that, globally, *TERTp* mutations occur in approximately 49.2% of HCC cases, with markedly higher frequencies observed in HCV-associated tumours (66.2%) compared to HBV-associated cases (31.6%) [10]. Additionally, mutations in *CTNNB1* exon 3, mainly amino acid substitutions at residues S29, D32, S33, G34, S37, T41, and S45, disrupt key phosphorylation sites, stabilizing β-catenin and thereby activating canonical Wnt/β-catenin signalling pathways that drive cellular proliferation and tumour progression [11]. *CTNNB1* exon 3 mutations, present in 23.1% of HCC cases worldwide, are similarly more prevalent in HCV-related HCC (30.7%) than in HBV-related tumours (12.8%) [10]. There is evidence of concordance between *TERTp* and *CTNNB1* mutations in HCC, suggesting a cooperative role in hepatocarcinogenesis, as studies indicate that their co-occurrence may promote tumour development and progression [12,13].

In Brazil, 10,598 deaths were attributed to HCC in 2022 [14], and an estimated 65% of cases are linked to chronic HCV infection [15]. Combined with the country’s pronounced admixed ancestry (European, African and Indigenous), this epidemiological profile creates a unique setting to examine how viral aetiology and host genetics shape the mutational landscape of HCC. Despite extensive international documentation, the prevalence and significance of *TERTp* and *CTNNB1* mutations remain largely unexplored among Brazilian patients.

Therefore, this study aims to analyse the prevalence of *TERTp* (C228T and C250T) and *CTNNB1* exon 3 mutations and evaluate *TERT* expression in tumour and non-tumour liver tissues from Brazilian patients, providing novel insights into the molecular pathogenesis of HCC in this genetically heterogeneous population.

## 2. Results

### 2.1. TERTp and CTNNB1 Exon 3 Mutations in Tumour Tissue Samples and Their Correlation with Clinicopathological Characteristics

Among the 42 HCC tumour tissue samples analysed, the *TERTp* C228T mutation was identified in 19 cases (45.2%). Patients harbouring this mutation were predominantly aged ≥60 years (68.4%), male (57.9%), with tumours < 5 cm in size (42.1%), and moderately differentiated (63.2%). Regarding underlying liver disease, 42.1% of *TERT*-mutated cases were associated with HCV infection, while no cases were linked to HBV. No statistically significant associations were found between *TERT* mutation status and clinicopathological variables (*p* > 0.05; Table 1).

Somatic mutations in exon 3 of *CTNNB1* were detected in 5 of the 29 HCC tumour samples (17.2%). These mutations were exclusively observed in patients aged ≥60 years (100%), predominantly male (60%), with tumours < 5 cm in size (60%), and moderate histological differentiation (60%). Most *CTNNB1*-mutated cases were associated with non-viral aetiologies (80%), while only 20% were linked to HCV infection and none were linked to HBV. Again, no statistically significant differences were observed between *CTNNB1* mutation status and the evaluated clinicopathological parameters (Table 1). It should be noted that the absence of statistically significant associations may be influenced by the small number of cases in each aetiological subgroup, which limits the power of the statistical analysis.

### 2.2. TERTp and CTNNB1 Exon 3 Mutations Across All Samples and Their Correlation with Liver Disease Tissue Types

Among the 85 liver tissue samples analysed, the *TERTp* C250T mutation was identified in only one case (1.2%), corresponding to HCC tumour tissue; the paired adjacent non-tumour tissue from this patient did not harbour the mutation. In contrast, the C228T mutation in the *TERTp* was detected in 23 of the 85 samples (27.1%), with distribution across tissue types as follows: 19 of 42 HCC tissues (45.2%), 4 of 21 cirrhotic tissues (19%), and none of the 22 hepatitis tissues. Statistically significant differences were observed between HCC and hepatitis tissues (*p* = 0.0001), hepatitis and cirrhotic tissues (*p* = 0.0485), and tumour versus non-tumour tissues overall (*p* = 0.0005). No statistically significant difference was found between HCC and cirrhotic tissues (*p* = 0.0542), though a trend was noted (Table 2). Overall, among the 42 HCC tissue samples analysed, *TERTp* mutations at positions C228T and C250T were identified in 47.6% of the samples (*n* = 19 and *n* = 1, respectively).

Somatic mutations in exon 3 of the *CTNNB1* gene were assessed in 67 liver tissue samples, revealing six mutated cases (9%). These included five HCC tissues, with amino acid substitutions at S29A (*n* = 1), S29F (*n* = 1), D32V (*n* = 1), and D32G (*n* = 2), and one cirrhotic tissue harbouring a T41P substitution. The overall mutation frequency was 17.2% (5/29) in HCC tissues, 5.6% (1/18) in cirrhotic tissues, and 0% in hepatitis tissues. Although *CTNNB1* exon 3 mutations appeared more frequent with advancing liver disease, no statistically significant correlation was observed across tissue types (*p* > 0.05; Table 2).

### 2.3. TERTp and CTNNB1 Exon 3 Mutations in Paired Samples and Their Correlation with Liver Disease Tissue Types

Analysis of paired tumour and adjacent non-tumour liver tissues revealed distinct mutation patterns. For the *TERTp* C228T mutation, 12 out of 26 patients (46.2%) exhibited the mutation exclusively in tumour tissue, while no mutations were detected solely in non-tumour tissue (*p* = 0.0001; Table 3). In addition, two patients (7.7%) exhibited the C228T mutation in both tumour and non-tumour tissues, while 12 patients (46.2%) had no detectable mutation in either tissue. Similarly, assessment of *CTNNB1* exon 3 mutations in paired samples showed that 4 of 24 patients (16.7%) harboured mutations only in tumour tissue, with no cases presenting mutations restricted to non-tumour tissue (*p* = 0.1092; Table 3). Additionally, one patient (4.2%) exhibited mutations in both tumour and non-tumour tissues, while 19 patients (79.2%) had no detectable mutation in either tissue.

These findings underscore that both *TERTp* and *CTNNB1* mutations preferentially localise to malignant tissue, although only *TERTp* mutations demonstrated a statistically significant enrichment when comparing paired tumour and non-tumour samples.

### 2.4. Association Between TERTp and CTNNB1 Exon 3 Mutations

Analysis of the association between *TERTp* C228T and *CTNNB1* exon 3 mutations revealed a notable overlap. Among the six samples harbouring *CTNNB1* exon 3 mutations, four (66.7%) also carried the C228T mutation in the *TERTp*. In contrast, only 13 of the 54 (24.1%) *CTNNB1* wild-type samples exhibited *TERTp* C228T. This association was statistically significant (*p* = 0.0485; Table 4).

### 2.5. TERT Gene Expression in Tumour and Non-Tumour Samples

**Figure 1:**
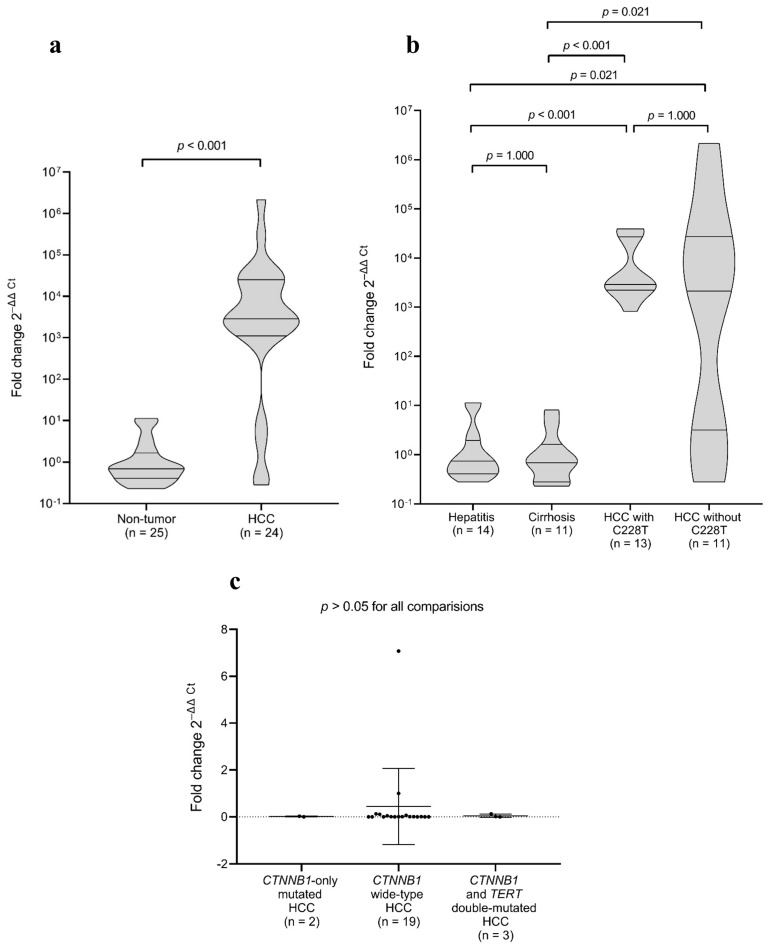
*TERT* gene expression according to tissue type and mutational status. (**a**) *TERT* mRNA expression levels in HCC tissues (*n* = 24) compared to non-tumour (cirrhosis and hepatitis) liver tissues (*n* = 25). *TERT* expression was significantly elevated in HCC tissues relative to non-tumour tissues (*p* < 0.001). (**b**) Comparison of *TERT* expression between HCC tissues harbouring the *TERTp* C228T mutation (*n* = 13) and those without the mutation (*n* = 11). No significant difference was observed between mutated and non-mutated HCCs (*p* = 1.000). Additionally, *TERT* expression between cirrhotic and hepatitis tissues was compared, showing no significant difference (*p* = 1.000). (**c**) *TERT* expression in HCC tissues stratified by *CTNNB1* mutation status: *CTNNB1*-mutated only, wild-type for *CTNNB1*, and harbouring both *CTNNB1* and *TERT* mutations. No statistically significant differences were detected among the groups (*p* > 0.05). *TERT* expression was measured through RT-qPCR using TaqMan assays, normalised to 18S rRNA and GAPDH. Relative quantification was calculated using the 2^−ΔΔCt^ method. Statistical significance was determined as *p* < 0.05.

*TERT* expression was significantly elevated in HCC tissues compared to non-tumour liver tissue samples diagnosed histologically as cirrhosis or hepatitis (*p* < 0.001; Figure 1a). No significant difference in *TERT* expression was observed between HCC samples harbouring the C228T mutation and those without this mutation (*p* = 1.000), providing evidence that *TERT* overexpression is a consistent feature of HCC, independent of C228T mutation status (Figure 1b). Additionally, the comparison between cirrhotic and hepatitis tissues revealed no significant difference in *TERT* expression levels (*p* = 1.000; Figure 1b). Further analysis showed no significant differences in *TERT* expression among HCC tissues with *CTNNB1* mutations only, wild-type *CTNNB1*, and those with both *CTNNB1* and *TERTp* mutations (*p* > 0.05; Figure 1c), suggesting that *CTNNB1* mutation status did not influence *TERT* expression levels in HCC tissues.

## 3. Discussion

In this study, we investigated the prevalence of *TERTp* and *CTNNB1* exon 3 mutations, as well as *TERT* gene expression, in HCC, cirrhotic, and hepatitis tissues from Brazilian patients. Our findings confirm and expand upon existing data, providing important insights into the molecular landscape of HCC in Latin American patients, a population underrepresented in global genomic studies.

*TERTp* and *CTNNB1* mutations have been reported to be significantly associated with older patient age, male sex, and smaller tumours (<5 cm) [12,16]. Although no statistically significant associations were found in our study, we also observed a higher frequency of these mutations in male and older patients, as well as in smaller, moderately differentiated tumours, aligning with the previous literature. In addition, *TERTp* mutations were frequent in HCV-related HCCs (42.1%) but absent in HBV-related cases. This pattern aligns with international data showing that *TERTp* mutations are strongly associated with HCV infection and are less common in HBV-related tumours, as confirmed by large multicentre studies [10,13,17]. The higher prevalence of *TERTp* mutations in HCV-associated HCC supports the hypothesis that chronic inflammation and oxidative stress from HCV infection promote genomic instability, favouring *TERTp* mutations [18]. In contrast, HBV-associated HCC shows more variable *TERTp* mutation rates, likely influenced by HBV genotypes, integration patterns, and cofactors such as aflatoxin exposure and metabolic disorders [19,20]. Although *TERT* promoter mutations are less frequent in HBV-related HCC compared to HCV-related cases, activation of the *TERT* gene in HBV infection can occur through alternative mechanisms, particularly via HBV DNA integration into the *TERT* locus. This integration may lead to aberrant *TERT* expression and has been reported as a frequent early event in HBV-associated hepatocarcinogenesis [20,21]. Regarding *CTNNB1* exon 3 mutations, they were markedly more prevalent in non-viral HCCs in our study, with a frequency of 80%. Consistently, *CTNNB1* mutations are well documented as being more frequent in non-viral HCCs (e.g., alcohol-related and MASLD) than in viral-related cases [12,22]. While *CTNNB1* mutations are key drivers of hepatocarcinogenesis, their prognostic value in established HCC remains controversial. Some studies link these mutations to less aggressive tumours, lower alpha-fetoprotein levels, and better differentiation [23,24,25], while others associate them with worse outcomes, such as vascular invasion, or find no significant impact on survival [26,27]. Although our findings did not reach statistical significance, likely due to sample size limitations, they reinforce the notion of distinct molecular pathways driving hepatocarcinogenesis according to underlying aetiology, underscoring the importance of considering viral status when interpreting the mutational landscape of HCC.

The 47.6% prevalence of *TERTp* mutations among the analysed Brazilian HCC cases is highly consistent with the 49.2% reported in a global series of 4133 HCC samples [10]. Our findings are also comparable to those from the USA (44.3%) [28], Germany (47.4%) [29], and Italy (50.4%) [30], despite slight regional differences. The C228T mutation in the *TERTp* was identified in 45.2% of HCC tissues, while the C250T mutation was detected in only one case (2.4%). This predominance of C228T over C250T is consistent with prior studies conducted across different geographic regions, where C228T accounts for approximately 90% of all *TERTp* mutations [10]. These data reinforce the notion that the C228T mutation generates a highly accessible binding site for ETS/TCF transcription factors, promoting telomerase activation more effectively than C250T [31]. Notably, we found that the *TERTp* C228T mutation was present in 19% of cirrhotic tissues, contrasting with earlier findings by Nault et al. (2013, 2014) [12,32], who reported no *TERTp* mutations in non-tumourous cirrhotic tissues of different aetiologies. This pattern of progressive increase, from 0% in hepatitis to 19% in cirrhosis and 45.2% in HCC, supports the hypothesis that *TERTp* C228T arises early in hepatocarcinogenesis, potentially within cirrhotic nodules prior to overt malignancy. These findings highlight the potential utility of the *TERTp* C228T mutation as a biomarker for enhanced surveillance and risk stratification in cirrhotic patients, warranting further investigation in longitudinal cohorts. It is worth noting that all tissue samples from this study underwent rigorous histopathological review to ensure accurate classification and exclude microscopic tumour infiltration, strengthening the validity of *TERTp* C228T detection in non-tumour cirrhotic tissues. While the clinical implementation of *TERTp* mutations screening is not yet established, our finding of C228T in nearly one-fifth of cirrhotic samples suggests a promising avenue for molecular surveillance in high-risk individuals. If validated in larger, prospective studies, *TERTp* mutational status could be incorporated into risk models and potentially guide personalised HCC monitoring strategies.

Regarding *CTNNB1* exon 3, mutations were identified in 17.2% of HCC tissue samples, a frequency comparable to the 23.1% prevalence reported in a global series of 5276 HCC cases [10]. This frequency is in line with previous reports from Iran (18.1%) [33], Taiwan (18.3%) [34], and France (19%) [35]. As expected, these mutations were nearly absent in cirrhotic and hepatitis tissues, corroborating the idea that *CTNNB1* mutations typically arise later in the carcinogenic cascade and are rare in precursor lesions [32,36].

*TERT* gene expression analysis revealed robust overexpression in HCC tissues compared to cirrhotic and hepatitis tissues (*p* < 0.001), independent of C228T mutation status. This aligns with findings from Nault et al. (2020), who demonstrated that although *TERT* is markedly upregulated in HCC, *TERTp* mutations are not the sole drivers of this overexpression, implicating additional regulatory mechanisms [37]. Consistent with this, we observed no significant difference in *TERT* expression between cirrhotic and hepatitis tissues, reinforcing that *TERT* upregulation is primarily linked to malignant transformation rather than to chronic liver injury alone. Furthermore, *TERT* expression did not vary significantly among HCC tissues stratified by *CTNNB1* mutation status, suggesting that β-catenin activation does not directly influence *TERT* transcription in this context. Our study also confirmed a significant association between *CTNNB1* and *TERTp* mutations, with 66.7% of *CTNNB1*-mutated HCCs also carrying the C228T mutation (*p* = 0.0485). While based on a small number of *CTNNB1*-mutated cases, the statistically significant co-occurrence with *TERTp* mutations supports previous reports [12,13,37,38] and suggests a potential synergistic role in hepatocarcinogenesis, which should be explored further in larger cohorts.

Beyond their role in tumourigenesis, *TERTp* and *CTNNB1* exon 3 mutations hold significant prognostic value. *TERTp* mutations correlate with aggressive HCC phenotypes, including reduced progression-free survival and increased recurrence rates after resection, independent of tumour stage or liver function [12,39]. Conversely, *CTNNB1*-mutated HCCs often exhibit distinct clinicopathological features (e.g., well-differentiated histology), though their prognostic impact remains debated [27,38]. Therapeutically, these mutations represent emerging targets for personalised approaches. Preclinical evidence suggests that *TERTp*-mutant HCCs may be more sensitive to Polo-like kinase 1 (PLK1) inhibitors due to telomere maintenance vulnerabilities and aberrant cell cycle regulation [40]. In contrast, *CTNNB1*-mutant tumours, characterised by an immune-excluded microenvironment and resistance to PD-1 blockade [41], may benefit from therapies targeting the Wnt/β-catenin pathway, such as the small-molecule inhibitor PRI-724, currently under investigation [42]. Integrating mutational profiling with these emerging therapies could refine risk stratification and expand treatment options for molecularly defined HCC subsets.

This study has several limitations. First, the relatively small sample size, particularly in subgroup analyses (e.g., *CTNNB1*-mutated HCCs), may limit statistical power and generalisability. Second, the samples were obtained from two tertiary centres in Southeastern Brazil, which may not fully capture the nationwide heterogeneity of HCC cases. Third, the use of Sanger sequencing, while robust for hotspot detection, has limited sensitivity for low-frequency variants, potentially underestimating mutation prevalence in heterogeneous tumour samples. Fourth, while we profiled mutational and gene expression patterns, functional assays were beyond the scope of this study and should be pursued in future research. Finally, due to the retrospective design of this study and incomplete long-term clinical records, we were unable to correlate *TERTp* and *CTNNB1* mutation status with patient survival, recurrence rates, or treatment outcomes. The absence of these clinical endpoints limits our ability to assess the prognostic value of these mutations in Brazilian HCC patients. Future prospective studies with standardised follow-up protocols are warranted to evaluate the impact of these molecular alterations on disease progression and therapeutic response. Despite these limitations, this is the first study to investigate *TERTp* and *CTNNB1* mutations in Brazilian HCC patients, providing valuable and novel insights into the molecular landscape of HCC in this population.

Importantly, while our findings reaffirm key international trends, they also underscore the need for larger, multicentric studies in Brazil, given the country’s unique viral and ethnic background. Despite the limited sample size, the mutation frequencies and patterns observed in this admixed population were largely consistent with global data, reinforcing the broad relevance of these molecular markers. Nevertheless, it remains unclear whether genetic ancestry could subtly influence the mutational landscape or modulate the interplay between tumour drivers in hepatocarcinogenesis. Future studies incorporating ancestry-informative markers and larger, geographically diverse cohorts will be essential to investigate potential ancestry-related differences, ensuring that biomarker-based surveillance strategies are equitable and effective across populations.

## 4. Materials and Methods

### 4.1. Study Population

Liver tissue specimens, either cryopreserved (*n* = 50) or formalin-fixed paraffin-embedded (FFPE) (*n* = 35), were obtained from two institutions in Brazil: the A.C.Camargo Biobank at A.C.Camargo Cancer Center, São Paulo State, and the Department of Pathology at Clementino Fraga Filho University Hospital, Rio de Janeiro State. Demographic and clinicopathological data (age, sex, histology and underlying liver disease aetiology) were abstracted from the corresponding medical records when available. However, for a small subset of cases, especially among older FFPE samples, complete clinical data were not retrievable due to limitations in archived records.

For analysis of the C228T and C250T hotspot mutations in *TERTp*, we examined 85 liver-tissue samples collected from 59 patients with HCC. Histopathological review classified each specimen into one of three disease stages: HCC tissue (*n* = 42), cirrhotic non-tumour tissue (*n* = 21), or hepatitis tissue without cirrhosis (*n* = 22). Paired tumour and adjacent non-tumour samples (cirrhosis or hepatitis) were available for 26 of these patients. Somatic mutations in exon 3 of *CTNNB1* were assessed in 67 liver specimens from 43 of the above patients. This subset comprised 29 HCC samples, 18 cirrhotic tissues, and 20 hepatitis tissues; paired tumour/non-tumour material was analysed from 24 patients. The smaller number of samples analysed for *CTNNB1* reflects differences in amplification efficiency, as a greater proportion of tissue specimens yielded high-quality PCR products for *TERTp* than for *CTNNB1*. All tissue samples were independently reviewed by experienced pathologists to ensure accurate histopathological classification. Particular attention was given to the distinction between tumour and adjacent non-tumour tissues, especially cirrhotic nodules, to prevent misclassification due to microscopic tumour infiltration. Only samples with well-demarcated histological features and no evidence of tumour cell contamination were included in the non-tumour groups.

All participants provided written informed consent for tissue collection, banking, and research use. The study was approved by the Ethics Committees of Clementino Fraga Filho University Hospital (approval 139/10), A.C.Camargo Cancer Center (2.485.580), and the Oswaldo Cruz Institute/FIOCRUZ (2.296.938).

### 4.2. DNA Extraction and PCR Amplification

DNA was isolated from cryopreserved liver tissue on the QIAsymphony platform (QIAGEN, Hilden, Germany) with the BC400-V7 protocol. DNA from FFPE tissue was extracted with the QIAamp DNA FFPE Tissue Kit (QIAGEN, Hilden, Germany) according to the manufacturer’s instructions.

To detect the C228T and C250T mutations in *TERTp*, a 270-bp (base pair) fragment was amplified with primers flanking both hotspots (forward 5′-GCCGGGCTCCCAGTGGATTCG-3′; reverse 5′-GCTTCCCACGTGCGCAGCAGGA-3′). Each 25 µL reaction contained 2 µL of genomic DNA and 1 U of Platinum Taq DNA Polymerase (Invitrogen, Waltham, MA, USA). Cycling parameters were as follows: 95 °C for 15 min; 42 cycles of 95 °C for 15 s, 63 °C for 15 s, and 72 °C for 45 s; followed by a final extension at 72 °C for 5 min, as previously described [43].

For exon 3 of *CTNNB1* (427 bp), PCR employed primers 5′-CCTGGCTATCATTCT GCTTTTC-3′ (forward) and 5′-TCAAAACTGCATTCTGACTTTCA-3′ (reverse) under the same reagent concentrations. Cycling conditions were as follows: 94 °C for 2 min; 35 cycles of 94 °C for 30 s, 58 °C for 30 s, and 72 °C for 1 min; final extension at 72 °C for 10 min, according to a published protocol [44].

Negative controls were included in both the extraction and PCR steps to monitor contamination. Amplicons were resolved on 1.5% agarose gels and visualised under UV illumination.

### 4.3. Nucleotide Sequencing

PCR products were purified using the QIAquick PCR Purification Kit (QIAGEN, Hilden, Germany) according to the manufacturer’s instructions. The *TERTp* and exon 3 of the *CTNNB1* gene were sequenced via direct Sanger sequencing using the BigDye Terminator v3.1 Cycle Sequencing Kit (Applied Biosystems, Waltham, MA, USA). Sequencing reactions were analysed on an ABI 3730xl automated sequencer (Applied Biosystems).

Sequence data were processed and analysed using MEGA11: Molecular Evolutionary Genetics Analysis version 11 [45]. The analysis focused on the *TERTp* hotspot mutations C250T and C228T, whereas amino acid substitutions at residues S29, D32, S33, G34, S37, T41, and S45 were assessed in *CTNNB1* exon 3.

### 4.4. RNA Extraction and TERT Reverse Transcription Quantitative PCR (RT-qPCR)

To evaluate the expression profile of the *TERT* gene, real-time PCR was performed to quantify *TERT* mRNA levels in the cryopreserved tissue samples. RNA extraction was automated using the QIAsymphony system (QIAGEN, Hilden, Germany) and the miRNA CT 400 V8 protocol from the QIAsymphony RNA Kit (QIAGEN, Hilden, Germany). In this protocol, total RNA was treated with the DNase enzyme provided in the kit during the purification process. The total RNA concentration was determined using a NanoDrop, and normalization was performed accordingly. RNA was reverse transcribed for first-strand cDNA synthesis using the SuperScript™ IV First-Strand Synthesis System (Invitrogen, Waltham, MA, USA) and random primers. Reverse transcription was performed by incubating the reaction mixture at 55 °C for 10 min, followed by inactivation at 80 °C for 10 min, according to the manufacturer’s instructions.

*TERT* expression was assessed using the TaqMan Applied Biosystems Gene Expression Assay (Hs00972656_m1) (Thermo Fisher Scientific, Waltham, MA, USA), and gene expression was normalised to the internal control 18S rRNA (Hs99999901_s1) and GAPDH (Hs99999905_m1) (Thermo Fisher Scientific, Waltham, MA, USA). RT-qPCR was performed under the following conditions: 50 °C for 2 min, initial denaturation at 95 °C for 10 min, followed by 45 cycles of 95 °C for 15 s and 60 °C for 1 min. The relative quantification of *TERT* mRNA was calculated using the 2^−ΔΔCt^ method [46], using ExpressionSuite v1.3 software (Applied Biosystems, Waltham, MA, USA). No reverse transcriptase (-RT) controls were loaded in parallel to the samples.

### 4.5. Statistical Analysis

Fisher’s exact test was used to assess associations between tissue type, clinicopathological variables, and the presence of *TERTp* C228T or *CTNNB1* mutations (R software version 4.4.1, R Foundation for Statistical Computing, Vienna, Austria). *TERT* expression was compared between HCC and non-tumour tissues using the Mann–Whitney U test and across hepatitis, cirrhosis, and HCC groups (stratified by mutation status) using the Kruskal–Wallis test followed by Dunn’s post hoc test. For multiple comparisons by *CTNNB1* mutation status, we employed one-way ANOVA with Holm–Sidak post hoc tests. Tests were chosen based on data distribution. *TERT* expression assays were run in technical duplicates. Additional statistical analyses were conducted in SigmaPlot version 14.0 (Systat Software, San Jose, CA, USA) and GraphPad Prism version 8.0.1 (GraphPad, La Jolla, CA, USA), with *p* < 0.05 considered statistically significant.

## 5. Conclusions

In conclusion, this study provides novel insights into the molecular landscape of HCC in Brazilian patients, demonstrating a high prevalence of *TERTp* mutations, particularly C228T, not only in tumour tissues but also in non-tumourous cirrhotic tissues. These findings strongly support the early emergence of *TERTp* mutations in hepatocarcinogenesis and highlight their potential as biomarkers for molecular surveillance and risk stratification in cirrhotic individuals, especially in regions with high HCV burden. Moreover, the significant co-occurrence of *TERTp* and *CTNNB1* exon 3 mutations suggests a possible synergistic role in tumour progression, pointing to distinct molecular pathways that could be exploited for therapeutic intervention. Despite the genetically admixed background of the Brazilian population, the mutational profiles observed were consistent with those in more homogeneous populations, reinforcing the broader applicability of these molecular alterations across diverse ethnic groups. Future studies involving larger, multicentric cohorts and functional analyses are essential to validate these findings, determine their prognostic value, and explore their utility in guiding personalised strategies for HCC detection, surveillance, and treatment.

## Figures and Tables

**Table 1:** Correlation between the presence of *TERTp* and *CTNNB1* exon 3 mutations in tumour tissue samples and the clinicopathological characteristics of patients.

Variables	*TERTp* C228T		*p*-Value	*CTNNB1* Exon3 Mutations		*p*-Value
	Negative *n* = 23 (%)	Positive *n* = 19 (%)		Negative *n* = 24 (%)	Positive *n* = 5 (%)	
Age (years)			0.7080			0.2981
≥60	11 (47.8)	13 (68.4)		15 (62.5)	5 (100)	
<60	6 (26.1)	4 (21.1)		6 (25)	0	
Unknown	6 (26.1)	2 (10.5)		3 (12.5)	0	
Sex			0.4384			1
Male	13 (56.5)	11 (57.9)		15 (62.5)	3 (60)	
Female	4 (17.4)	6 (31.6)		6 (25)	2 (40)	
Unknown	6 (26.1)	2 (10.5)		3 (12.5)	0	
Aetiology			0.07872			1
HBV	3 (13)	0		0	0	
HCV	4 (17.4)	8 (42.1)		6 (25)	1 (20)	
Non-viral	16 (69.6)	11 (57.9)		18 (75)	4 (80)	
Tumour size			0.4795			0.6146
≥5 cm	10 (43.5)	7 (36.8)		12 (50)	2 (40)	
<5 cm	6 (26.1)	8 (42.1)		7 (29.2)	3 (60)	
Unknown	7 (30.4)	4 (21.1)		5 (20.8)	0	
Tumour differentiation			0.7854			0.3377
Well	0	1 (5.3)		1 (4.2)	0	
Moderately	14 (60.9)	12 (63.2)		17 (70.8)	3 (60)	
Poorly	2 (8.7)	1 (5.3)		0	1 (20)	
Unknown	7 (30.4)	5 (26.3)		6 (25)	1 (20)	

**Table 2:** Correlation between *TERTp* and *CTNNB1* exon 3 mutation frequencies and liver disease tissue types across all samples.

Mutation	Tissue Type (*n*, %)			*p*-Value			
	Non-Tumour		Tumour	Tumour X Non-Tumour	Hepatitis X Cirrhosis	HCC X Hepatitis	HCC X Cirrhosis
	Hepatitis	Cirrhosis	HCC				
*TERTp* C228T	0/22 (0)	4/21 (19)	19/42 (45.2)	0.0005	0.0485	0.0001	0.0542
*CTNNB1* exon 3	0/20 (0)	1/18 (5.6)	5/29 (17.2)	0.0776	0.4737	0.0704	0.3839

**Table 3:** Correlation between *TERTp* and *CTNNB1* exon 3 mutation frequencies and liver disease tissue types in paired samples.

Mutation	Paired Tissue (*n*, %)		*p*-Value
	Tumour ^1^	Non-Tumour ^1^	
*TERTp* C228T	12/26 (46.2)	0/26 (0)	0.0001
*CTNNB1* exon 3	4/24 (16.7)	0/24 (0)	0.1092

^1^ Mutations detected exclusively in this tissue type.

**Table 4:** Co-occurrence of *TERTp* C228T and *CTNNB1* exon 3 mutations.

Mutation	*CTNNB1* Mutations Positive	*CTNNB1* Mutations Negative	*p*-Value
*TERTp* C228T positive	4/6 (66.7%)	13/54 (24.1%)	0.0485
*TERTp* C228T negative	2/6 (33.3%)	41/54 (75.9%)	

## Data Availability

The data supporting the findings of this study are available from the corresponding author upon reasonable request. These data are not publicly available due to privacy and ethical restrictions.

## Abbreviations

The following abbreviations are used in this manuscript:

bp	base pair
CTNNB1	catenin beta 1
FFPE	formalin-fixed paraffin-embedded
HBV	hepatitis B virus
HCC	hepatocellular carcinoma
HCV	hepatitis C virus
MASLD	metabolic dysfunction-associated steatotic liver disease
NAFLD	non-alcoholic fatty liver disease
PLK1	polo-like kinase 1
RT-qPCR	reverse transcription quantitative PCR
TERTp	telomerase reverse transcriptase promoter
